# Communicable disease mortality trends and characteristics of infants in rural China, 1996–2015

**DOI:** 10.1186/s12889-020-08486-y

**Published:** 2020-04-06

**Authors:** Ke Wang, Liangcheng Xiang, Leni Kang, Lei Miao, Qi Li, Xiaohong Li, Jun Zhu, Yanping Wang, Yan Huang, Chunhua He

**Affiliations:** 1 National Office for Maternal and Child Health Surveillance of China, Department of Pediatrics, West China Second University Hospital, Sichuan University, No. 17, Section 3 South Renmin Road, Chengdu, 610041 Sichuan China; 2 Key Laboratory of Birth Defects and Related Diseases of Women and Children (Sichuan University), Ministry of Education, Chengdu, China; 3 Department of Obstetrics, West China Second University Hospital, Sichuan University, Chengdu, China

**Keywords:** Communicable disease, Regional characteristics, Time trend, Infant mortality rate

## Abstract

**Background:**

More attention should be paid to communicable disease-specific infant mortality rate (CD-IMR) in rural China. However, few studies have examined specific geographic patterns and trends in CD-IMR in these areas. Our aims were to assess the epidemiological distribution and trends in CD-IMR in rural China for the period 1996–2015.

**Methods:**

We used data from China’s Under-5 Child Mortality Surveillance System (U5CMSS). The time trends in communicable disease-specific IMR (CD-IMR) were assessed by Poisson regression model, and the proportion of total infant deaths due to communicable disease was assessed by the Cochran Armitage trend test. Differences in CD-IMR among and within geographic regions were assessed for significance using the Cochran–Mantel–Haenszel test.

**Results:**

The overall CD-IMR fell by 86.0% from 1444.3 to 201.5 per 100,000 live births in rural mainland China from 1996 to 2015. The proportion of total infant deaths related to communicable disease fell from 33.4 to 19.7%. Using eastern rural areas as the reference, rate ratios (RRs) of IMR due to all communicable diseases ranged between 1.7 and 3.1 in central rural areas and between 4.4 and 9.8 in western areas during the four study intervals. Acute respiratory infection (ARI) accounted for 71% of deaths, followed by diarrhea and septicemia.

**Conclusions:**

IMR due to communicable disease remains a major public health issue. ARI is the leading cause of mortality, followed by diarrhea. A regional gap remains in the risk of infant exposure to communicable disease in rural China. More attention should be paid to western rural areas.

## Background

In 2015, China and other nations entered a new phase of development underpinned by the Sustainable Development Goals (SDGs). SDG 3 proposes to end preventable death of newborns and children under 5 years of age and to end epidemics or communicable diseases such as AIDS, tuberculosis, malaria and water-borne diseases by 2030 [1].

Over the last 20 years, China has experienced a substantial decline in under-five mortality. However, the threat of communicable diseases persists for children under 5 years, with communicable diseases remaining a leading source of morbidity and mortality. In 2015, approximately 19% of deaths among children 5 years of age were due to communicable disease [2]. Deaths caused by communicable disease can be avoided through prevention and treatment, education, immunization campaigns, and reproductive health care. It is particularly important to target such prevention and intervention efforts at infants, since infant deaths account for 75% of deaths among children under 5 years of age in China [3]. Thus, reducing infant deaths due to communicable disease may be the most effective way to achieve SDG 3.

Between 1996 and 2015, IMR fell in China from 36.0 [4] to 8.1 per 1000 live births [5], which is lower than in most developing countries [6]. However, great disparities in IMR remain because of geographical differences and imbalanced economic development. For example, during 1996–2015, the mortality rate due to pneumonia among children under 5 years of age was higher in rural areas than in urban areas [7]. It is likely that a similar disparity holds for infants. Therefore, more attention should be paid to communicable disease-specific IMR (CD-IMR) in rural China. However, few studies have examined specific geographic patterns and trends in communicable disease-specific IMR in these areas.

The present study examined these patterns and trends for the period 1996–2015, with the aim of providing evidence for effective interventions and child health policy to improve infant health in rural China and potentially other developing countries.

## Methods

### Data sources

Data in this study were taken from the Under-5 Child Mortality Surveillance System (U5CMSS), established in the 1990s by the Chinese Ministry of Health. This population-based surveillance system collects data from 31 provinces in Mainland China (excluding Hong Kong, Macao and Taiwan), which established 123 surveillance sites (districts/counties) before 2009, and increased to 334 sites from 2009 onwards. U5CMSS came to its current structure in 2013, covering 334 sites with a surveillance population of 47.1 million. The mainland of China was divided into three geographic regions (i.e., eastern, central, and western regions) based on the criteria from the National Development and Reform Commission of China, and each region was further stratified into urban and rural areas, according to the criteria used in the National Health Services Survey and the administrative division codes published by the National Bureau of Statistics. The geographical distribution of surveillance sites in the three geographic regions of Mainland China has been reported [2]. More detailed information about sampling methods, subjects, data collection, and quality control in U5CMSS can be found in previous studies [7, 8] . In this study, U5CMSS data are available annually for the period 1996–2015.

### Verification of causes of death

Cause of death was determined from death certificates in the case of children who died in hospital, from hospital-reported medical diagnoses in the case of children who used healthcare before death, or from verbal autopsies in the case of no medical record available. Doctors from township hospitals and community health service centers conducted verbal autopsies and deduced causes of death based on disease definitions in the *Zhu Futang Practice of Pediatrics*. Causes of all deaths were confirmed by pediatricians at maternal and child health institutes at the county, municipal and provincial levels.

All deaths determined to be caused by communicable disease were included in the present study. Communicable diseases were classified according to the International classification of Diseases-10, including respiratory infection (ARI; H65-H66, J00-J22, J85, P23), diarrhea (A00-A09), septicemia (P36, A40–41), meningitis/encephalitis (A39, A83, A84-A87, G00, G03, G04) and other communicable diseases (A10-A32, A36, A38, A42-A82, A88-B04, B06-B19, B25-B49, B55-B99, N30, N34, N39.0, N70-N73, U04).

### Statistical analysis

Data from 123 representative sampling sites were used for analysis during 1996–2008 and data from 336 sites were used for analysis during 2009–2015. We defined CD-IMR as the number of deaths from communicable disease divided by the number of live births during the same time period. The IMR was adjusted by the 3-year moving average under-reporting rate [9] and expressed as deaths per 100,000 live births. The rate was separated by geographic region and weighted by the proportion of different geographic region population from the National Census to obtain the overall estimation.

The annual number of infants who died of specific-type communicable disease was relatively low. To obtain a reliable estimation, the entire study period of 1996–2015 was divided into four intervals: 1995–2000, 2001–2005, 2006–2010 and 2010–2015. Poisson regression was used to estimate the but Average Annual Percent Change (AAPC) in order to evaluate trends in CD-IMR at the national and regional levels. CD-IMRs between regions were compared through rate ratio (RR) and the associated 95% confidence interval (CI), which were calculated using the Cochran-Mantel-Haenszel (CMH) test [10]. The trend in proportion of total infant deaths due to communicable disease was analyzed using the Cochran–Armitage trend test [11]. All statistical analyses were performed using SAS 9.4. Statistical significance was assessed using two-tailed tests at an alpha level of 0.05.

## Results

**Fig. 1:**
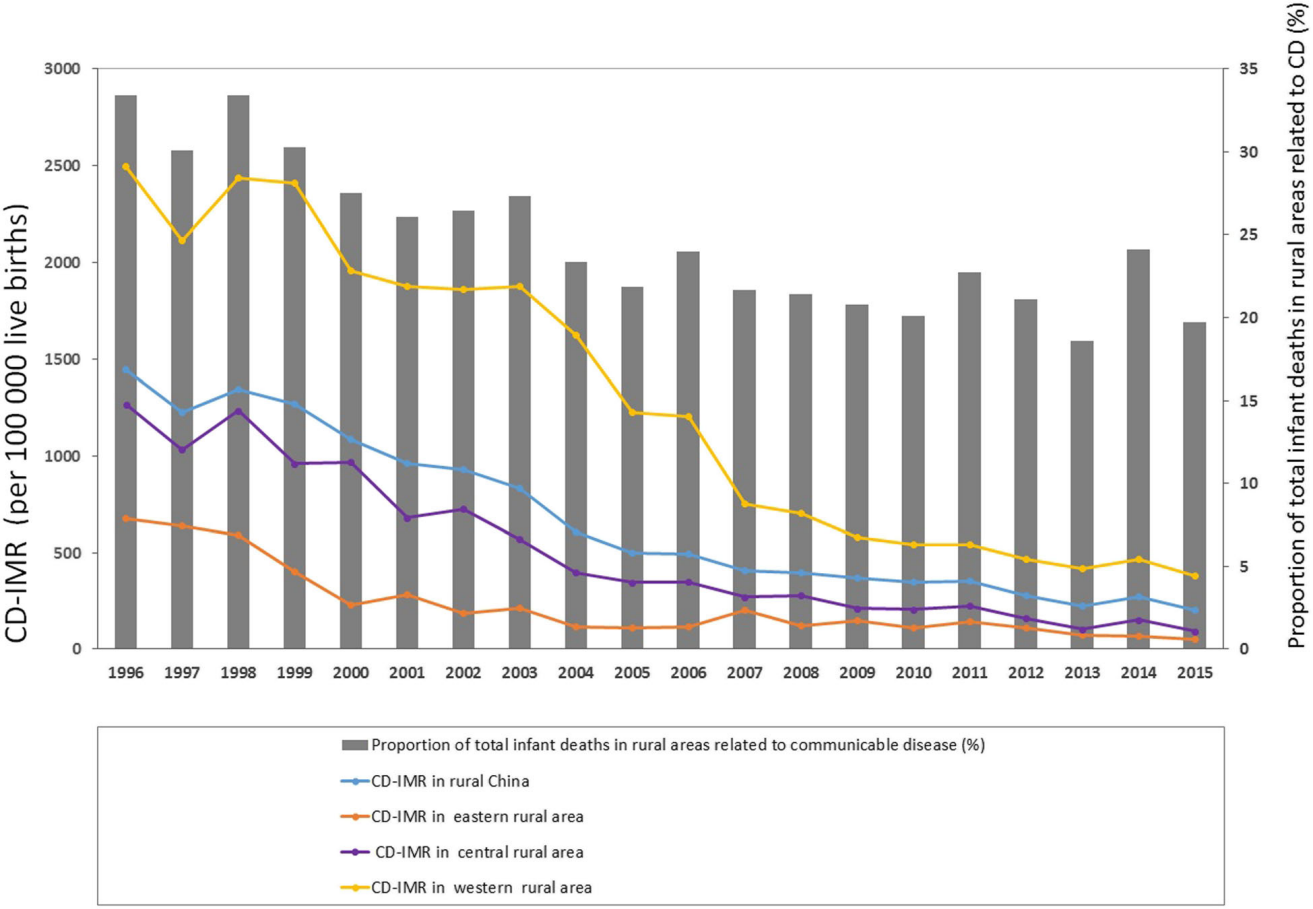
Communicable disease (CD)-related IMR (per 100,000 live births) (left *y*-axis) and the proportion of total infant deaths in rural China due to communicable disease (right *y*-axis), 1996–2015

Between 1996 and 2015, U5CMCC surveillance sites recorded 2,244,503 live births. The East, Middle and West China accounted for 21.7, 41.3 and 37.0% of total live births, respectively. Among them, there were 10,145 infant deaths due to communicable diseases. The East, Middle and West China accounted for 7.7, 28.6 and 63.7% of total infant mortality, respectively (Table S1). CD-IMR fell by 86.0% in rural mainland China, from 1444.3 to 201.5 per 100,000 live births. The annual rate of decline was 10.5% (*P* < 0.001). The proportion of communicable disease caused deaths in total infant mortality also decreased from 33.4% in 1996 to 19.7% in 2015. This proportion decreased continuously over the last 20 years of the study period, with fluctuation during the last 10 years. The CD-IMR decreased in the three regions of rural China as well as in rural regions across the entire mainland. The IMR was highest in western rural areas and lowest in eastern rural areas (Fig. 1).

**Fig. 2:**
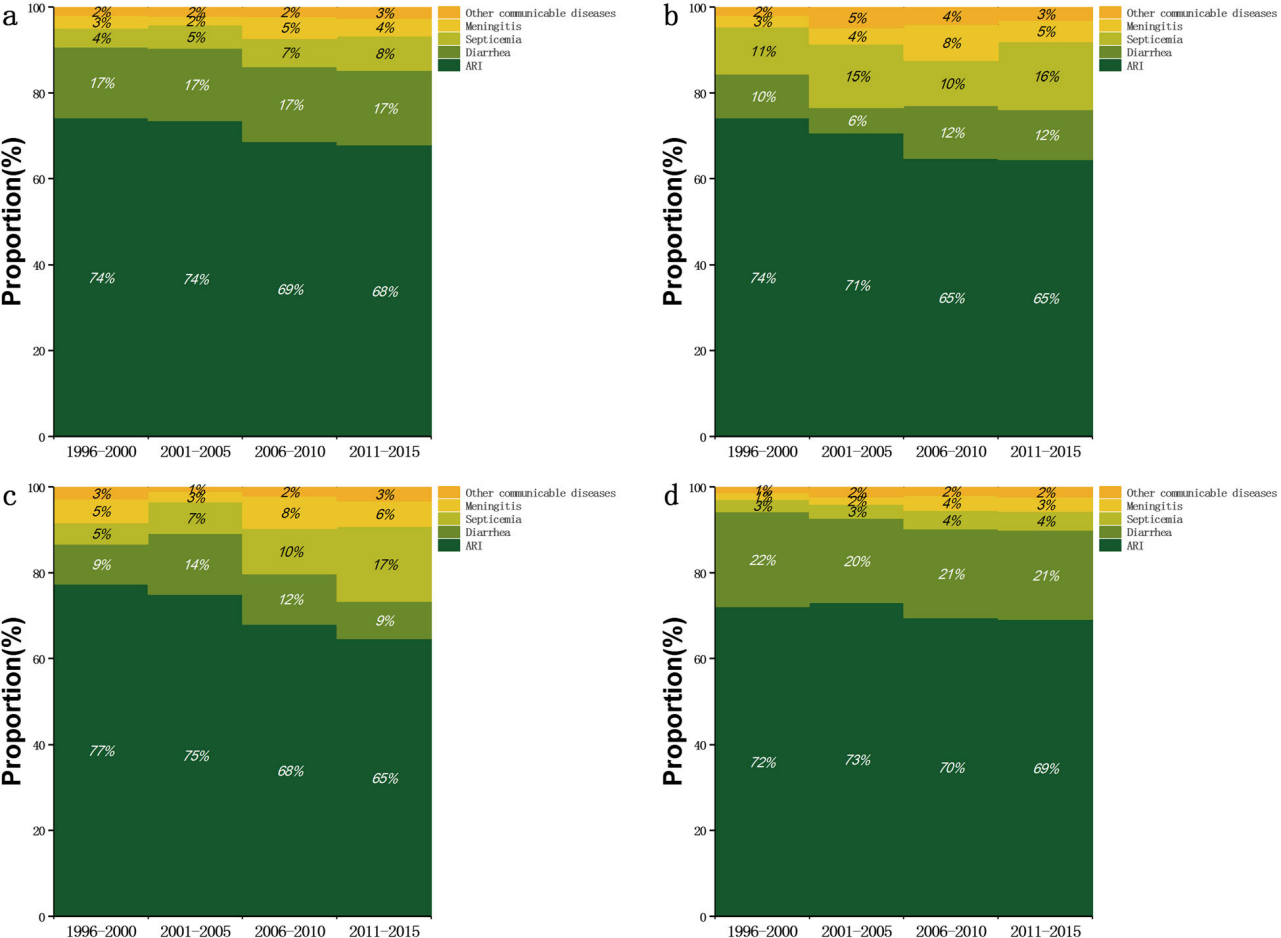
Proportion of communicable disease-related deaths due to specific diseases (**a**) nationwide in rural regions, **b** in eastern rural regions, **c** in central rural regions or (**d**) in western rural regions for four intervals between 1996 and 2015

During the study period, ARI, diarrhea and septicemia were the top three communicable diseases causing infant mortality in eastern, central and western rural areas of China, as well as in rural regions across the entire Mainland China. Nationwide, ARI, diarrhea and septicemia accounted for ~ 71%, ~ 17 and 7% of infant deaths due to communicable disease in rural areas, and these proportions did not vary significantly over the study period. The proportion of ARI and its time trend in rural areas of the three regions were similar to the results in rural areas across the Mainland China. The second most frequent communicable disease caused infant mortality was septicemia in eastern rural areas, and diarrhea in central and western rural areas. Diarrhea contributed to 6–12% of communicable disease-related infant deaths in eastern rural areas, 9–14% in central rural areas and 20–22% in western rural areas (Fig. 2).

**Fig. 3:**
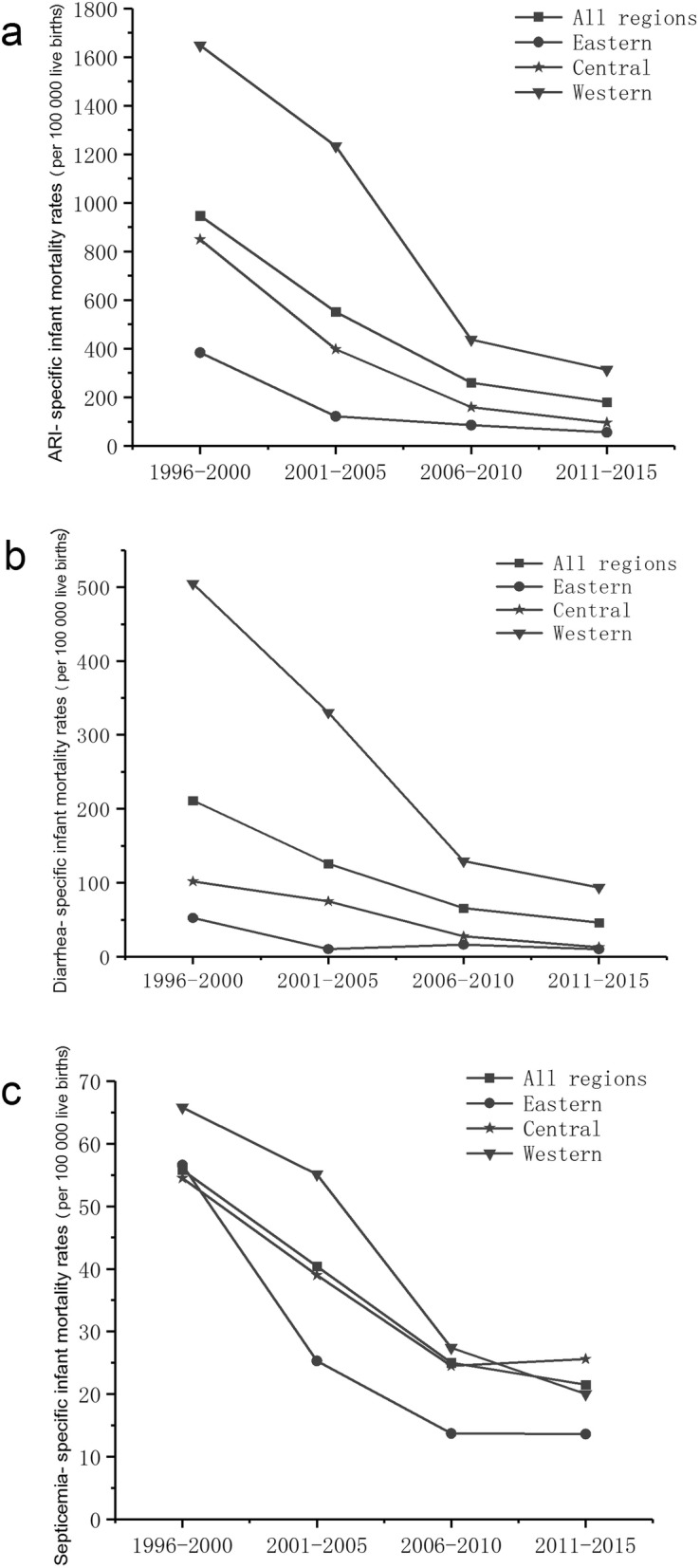
Communicable disease-specific infant mortality rates in nationwide rural regions, eastern rural regions, central rural regions and western rural regions between 1996 and 2015. **a** ARI-specific infant mortality rate, **b** Diarrhea-specific infant mortality rate, **c** Septicemia-specific infant mortality rate

During 1996–2000 and 2011–2015, ARI-, diarrhea- and septicemia-specific IMRs decreased significantly in rural areas across mainland China by 81.0, 78.2 and 61.4%, respectively. These decreases accounted for 75.8, 16.3 and 3.4% of the overall decrease in CD-IMR. These three diseases caused IMR also decreased in eastern, central and western rural areas, mirroring the trend in total CD-IMR. Significant disparities among disease specific IMRs were observed among rural areas in different regions. Generally, the highest rates were observed in western rural areas and the lowest in eastern rural areas (Fig. 3).

Using eastern rural areas as the reference, RRs of CD-IMR ranged between 1.7 and 3.1 in central rural areas and between 4.4 and 9.8 in western areas during the four study intervals. RRs peaked in 2001–2005, which RR was 3.1 for central areas and 9.8 for western areas. These RRs decreased to 1.7 and 5.2 in 2011–2015, respectively. The time trend of RRs for IMR due to ARI and diarrhea was similar to the time trend of all communicable diseases. Differences in septicemia- or meningitis-specific IMR in rural areas of different regions did not show obvious correlation with ARI- or diarrhea-specific IMR (Table 1).

**Table 1:** Rate ratio -based comparison of communicable disease-specific IMRs between different rural regions in China

Rural regions and time period	Rate ratio of communicable disease-specific IMRs between different regions				
	All diseases	ARI	Diarrhea	Septicemia	Meningitis
Central vs. Eastern					
1996–2000	2.1 (1.9, 2.4)	2.2 (1.9, 2.6)	1.9 (1.3, 2.9)	1.0 (0.6, 1.5)	4.2 (2.0, 8.9)
2001–2005	3.1 (2.5, 3.8)	3.3 (2.6, 4.2)	7.8 (3.4, 17.9)	1.5 (0.9, 2.7)	2.1 (0.7, 6.2)
2006–2010	1.8 (1.5, 2.1)	1.9 (1.5, 2.3)	1.7 (1.0, 2.7)	1.8 (1.1, 3.1)	1.6 (0.9, 2.9)
2011–2015	1.7 (1.5, 2.0)	1.7 (1.4, 2.1)	1.3 (0.8, 2.1)	1.9 (1.3, 2.7)	2.1 (1.1, 4.2)
Western vs. Eastern					
1996–2000	4.4 (3.9, 5.0)	4.3 (3.7,5.0)	9.5 (6.6, 13.8)	1.2 (0.7, 1.8)	2.3 (1.0, 5.1)
2001–2005	9.8 (8.1, 12.0)	10.2 (8.1, 12.9)	34.6 (15.4, 77.8)	2.2 (1.2, 3.9)	4.4 (1.5, 12.8)
2006–2010	4.8 (4.1, 5.6)	5.1 (4.2, 6.2)	7.9 (5.0, 12.2)	2.1 (1.2, 3.5)	2.0 (1.1, 3.6)
2011–2015	5.2 (4.5, 6.0)	5.6 (4.7, 6.7)	9.4 (6.2, 14.2)	1.5 (1.0, 2.2)	3.7 (1.9, 7.2)

## Discussion

In the period of 1996–2015, the infant mortality due to communicable diseases has decreased significantly. Our data suggests that the overall CD-IMR in rural Mainland China has declined at an annual rate of 10.4%, from 1444.3 per 100,000 live births in 1995 to 201.5 per 100,000 live births in 2015. This decline may be attributed to rapid economic growth, reflected in the 752.0% increase in domestic product per capita during the same period [12, 13]. This growth has driven improvement in child health service infrastructure, pediatrician training, transportation condition, and education for girls. In parallel, children’s accessibility to health services has improved. For example, the percentage of children younger than 3 years covered by systematic health management expanded from 61.4% in 1996 to 87.0% in 2012 [14] (Additional file 1: Fig. S1). Implementation of major policies and programs has also contributed to the decline in under-5 mortality. For example, the New Rural Cooperative Medical System implemented in 2003 has reduced the rural residents’ expenses on hospitalization and improved the utilization of inpatient services for communicable diseases among children. Government programs in the 1990s to improve drinking water quality and lavatory hygiene may have helped reduce diarrhea-specific IMR. Several studies suggested that the program of integrated management of childhood illness, implemented in 1998, has contributed to the reduction in under-5 mortality related to communicable diseases [15–19]. It is likely that the reduction also reflects the success of other public health programs, such as programs to vaccinate infants against communicable diseases such as tuberculosis, syphilis, hepatitis B virus, and maternal-child transmission of human immunodeficiency virus (HIV).

Communicable diseases are easier to prevent and control than congenital and chronic diseases. This likely helps explain the drastic reduction of CD-IMR in our study. Nevertheless, the proportion has remained relatively high at around 20%, over the last 10 years. Health departments and the public health community in general should realize that communicable disease remains a leading cause of mortality among children younger than 1 year.

Our results indicate substantial regional differences in communicable disease-specific IMRs. Relative to eastern regions, the adjusted RR of IMR in western regions was more than 4 throughout the study period, while the adjusted RR in central regions was 1.7–3.1. These findings are consistent with results of previous work [20] and with the broader literature on the persistence of children’s health disparities in developed and underdeveloped countries [21–25]. Such persistence may reflect socioeconomic inequalities and poor healthcare [26–29]. In China, eastern regions are generally the most developed, followed by central and western regions. Unequal economic development may contribute to the differences in CD-IMRs observed in the present study. The parents of many children living in rural areas, especially in western rural regions, have only basic education, so they may lack the information and knowledge necessary to prevent and control communicable diseases [30]. They may also be unable to accurately recognize or assess communicable disease risk on their own, thereby increasing the possibility of mortality. Another risk factor for infant mortality in rural regions may be inadequate healthcare resources [31]. A national healthcare survey in 2012 reported 9.56 healthcare workers per 1000 in township hospitals and 3.86 healthcare workers per 1000 in rural clinics in eastern regions, and the corresponding numbers in western regions were 7.41 and 3.28 [32]. We observed a greater reduction in CD-IMR in eastern rural regions than in central or western rural regions in 1996–2006, consistent with previous work [7]. Several factors may explain this geographic difference, including a more developed economy, greater integration of children into the healthcare system, higher per capita net income of rural households, modernized pediatric infrastructure and transportation, and higher education level for women. In 2006–2015, the gap between eastern rural regions and rural regions in the rest of the country narrowed. The improved situation in central and western regions may reflect contributions from the integrated management of childhood diseases; the Great Western Development Strategy, which included construction of health infrastructure and pediatrician capacity-building and of course overall economic development.

Throughout the study period, ARI accounted for 68–74% of communicable disease-related infant mortality. This is consistent with previous study [33] reporting that ARI such as pneumonia is the leading cause of mortality among children younger than 1 year with immature respiratory and immune systems. The proportion of CD-IMR due to diarrhea was 17% in China from 1996 to 2015. The relatively low proportion of deaths due to diarrhea likely reflects the success of a Chinese government program in the 1990s to improve drinking water quality and lavatory hygiene. This program increased the percentage of the population with access to clean water from 43.2 to 74.6% between 1995 and 2012, and the percentage of the population with access to sanitary latrines increased from 39.8 to 71.7% between 1999 and 2012 [34]. However, a unique insight that emerges from our analysis is that, throughout the study period, the rates of mortality due to ARI or diarrhea were higher in central and western rural regions of China than in eastern rural regions. We also found septicemia and meningitis to be the relatively important causes of death related to communicable disease, and the rates of such deaths were higher in western rural regions. Therefore, implementing more child survival policies, resources, and programs, especially in central and western regions, should be a priority in order to reduce avoidable deaths. For example, programs such as health education should be strengthened to help parents recognize and prevent ARI, diarrhea, septicemia and meningitis early. A disease monitoring network should be established for collecting data on disease-related deaths and risk factors. Fast-track pathways should be established for treatment of disease and for referral of critically ill under-five children.

Besides, the new surveillance sites were established in 2007 with rigorous training for the recruited employee. After 2 years of test run, U5CMCC assessed the data quality from the new sites in 2009. After meeting U5CMCC standard, the data from new sites were used to analyze CD-IMR. The data from all surveillance sites maintain a good representation at national and regional level. We compared the mortality rate from the surveillance sites established before 2009 with mortality rate from all surveillance sites and found statistically significant differences in rates between the two data sets in 2012 and 2015 (see the Table S2 for yearly 261 comparison), however the entity of the differences allow for the use of the data.

The results in this study should be interpreted with caution. Since the study is based on a sample survey, the results have some sampling error compared to the census. Communicable diseases were diagnosed based mainly on verbal autopsy or clinical symptoms, which may increase risk of misdiagnosis. Nevertheless, 87% of the infants included in our analysis received medical treatment before death and so were diagnosed by specialists in healthcare facilities.

## Conclusion

The rate of mortality due to communicable disease declined among infants in rural areas of China from 1996 to 2015. This likely reflects the country’s rapid economic growth as well as its implementation of policies and programs to eliminate poverty and improve child health. Nevertheless, infant mortality due to communicable disease remains a major public health problem. Communicable disease-specific IMR is higher in western rural regions than in eastern rural regions. In rural regions, ARI remains the leading cause of mortality among infants, followed by diarrhea and septicemia. In China, expansion of public health policies, capital investment, health education, and access to medical resources--especially in western rural regions--should be a priority for reducing and ending preventable deaths from communicable disease in infants.

## Supplementary information

**Additional file 1:Figure S1.** (a) Per capita net income of rural residents (RMB/yr) and ARI-specific infant mortality rate, (b) Proportion of children under 3 covered by systematic health management and ARI-specific infant mortality rate.

**Additional file 2:Table S1.** The basic data of infant communicable disease mortality in rural China, 1996–2015.

**Additional file 3:Table S2.** The comparison of communicable disease-specific IMR between 123 and 334 surveillance sites.

## Data Availability

The datasets used and/or analyzed during the current study are available from the corresponding author on reasonable request.

## Abbreviations

IMR: Infant mortality rate
CD-IMR: Communicable disease-specific IMR
ARI: Acute respiratory infection
SDGs: Sustainable Development Goals
U5CMSS: Under-5 Child Mortality Surveillance System
RR: Rate ratio
CI: Confidence interval
CMH: Cochran-Mantel-Haenzel
