# Supplementary material for: Communicable disease mortality trends and characteristics of infants in rural China, 1996–2015
Source: BMC Public Health. 2020 Apr 6;20:455. doi: 10.1186/s12889-020-08486-y (PMC7137429; doi:10.1186/s12889-020-08486-y)
Supplement: Supplementary file 1 — Additional file 1:Figure S1. (a) Per capita net income of rural residents (RMB/yr) and ARI-specific infant mortality rate, (b) Proportion of children under 3 covered by systematic health management and ARI-specific infant mortality rate. [file 12889_2020_8486_MOESM1_ESM.docx]

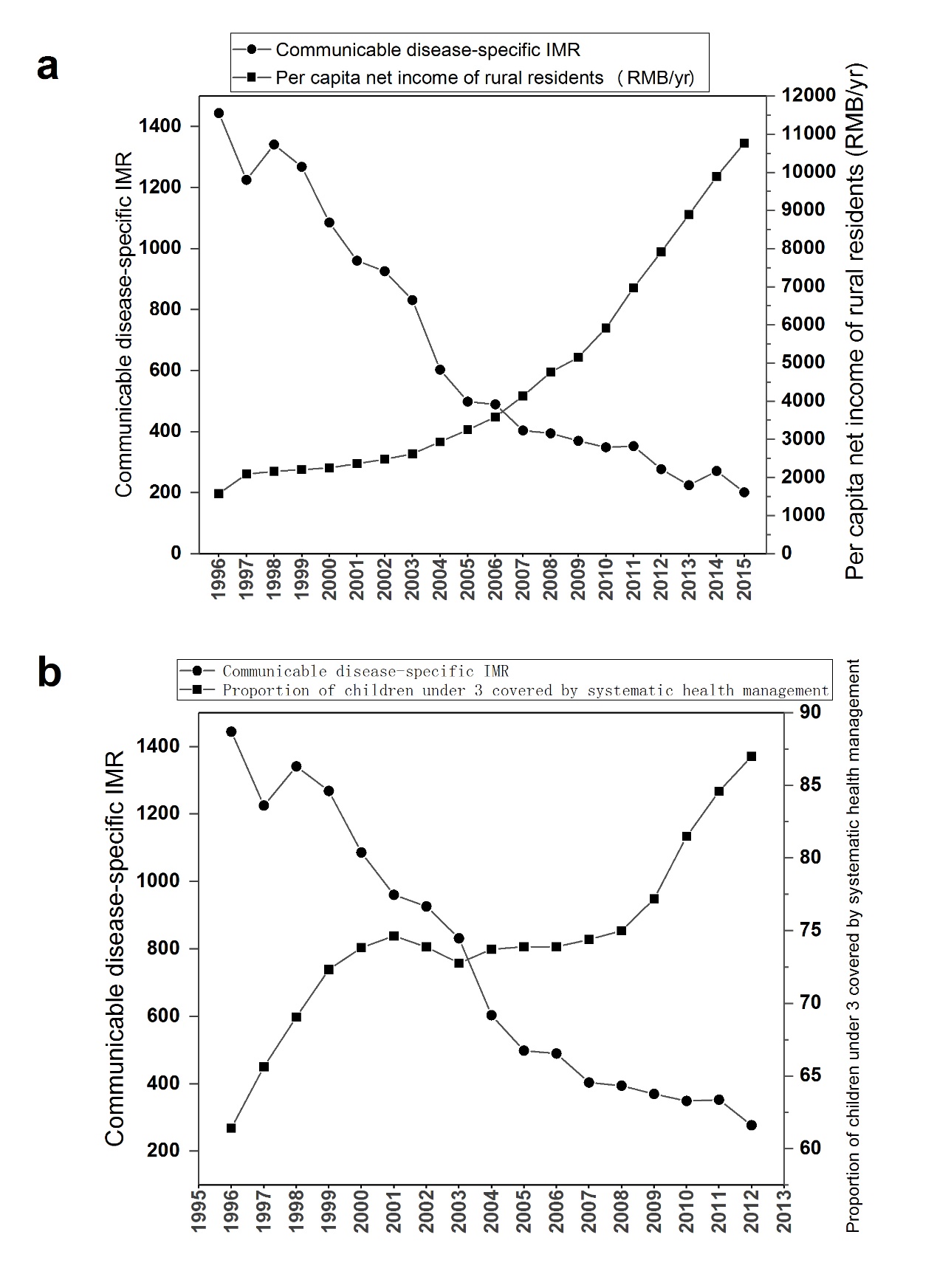


**Figure S1.** (a) Per capita net income of rural residents (RMB/yr) and ARI-specific infant mortality rate, (b) Proportion of children under 3 covered by systematic health management and ARI-specific infant mortality rate.
