# Supplementary material for: Communicable disease mortality trends and characteristics of infants in rural China, 1996–2015
Source: BMC Public Health. 2020 Apr 6;20:455. doi: 10.1186/s12889-020-08486-y (PMC7137429; doi:10.1186/s12889-020-08486-y)
Supplement: Supplementary file 2 — Additional file 2:Table S1. The basic data of infant communicable disease mortality in rural China, 1996–2015. [file 12889_2020_8486_MOESM2_ESM.docx]

Table S1 The basic data of infant communicable disease mortality in rural China, 1996-2015.

|  | Eastern rural regions | | | | | Central rural regions | | | | | Western rural regions | | | | |
| --- | --- | --- | --- | --- | --- | --- | --- | --- | --- | --- | --- | --- | --- | --- | --- |
|  | Liver births | Communicable disease deaths | ARI-specific deaths | Diarrhea-specific deaths | Septicemia-specific deaths | Liver births | Communicable disease deaths | ARI-specific deaths | Diarrhea-specific deaths | Septicemia-specific deaths | Liver births | Communicable disease deaths | ARI-specific deaths | Diarrhea-specific deaths | Septicemia-specific deaths |
| 1996 | 12800 | 86 | 65 | 10 | 6 | 21154 | 268 | 206 | 33 | 13 | 17629 | 440 | 290 | 109 | 18 |
| 1997 | 11915 | 76 | 57 | 5 | 13 | 19492 | 201 | 162 | 20 | 7 | 16492 | 349 | 261 | 69 | 9 |
| 1998 | 10987 | 65 | 48 | 9 | 6 | 18664 | 231 | 181 | 25 | 6 | 14859 | 362 | 263 | 85 | 9 |
| 1999 | 10379 | 42 | 32 | 6 | 5 | 18126 | 174 | 138 | 16 | 7 | 14878 | 359 | 279 | 66 | 9 |
| 2000 | 10457 | 24 | 15 | 0 | 3 | 18438 | 178 | 129 | 3 | 20 | 15137 | 296 | 209 | 70 | 7 |
| 2001 | 10323 | 29 | 22 | 2 | 3 | 19036 | 130 | 106 | 11 | 8 | 14456 | 271 | 216 | 43 | 7 |
| 2002 | 10732 | 20 | 14 | 1 | 3 | 19850 | 144 | 110 | 20 | 7 | 13810 | 257 | 199 | 40 | 9 |
| 2003 | 11727 | 25 | 18 | 1 | 3 | 20084 | 114 | 81 | 19 | 10 | 13528 | 254 | 180 | 54 | 7 |
| 2004 | 14683 | 17 | 13 | 0 | 2 | 23278 | 92 | 63 | 16 | 11 | 14216 | 231 | 170 | 46 | 8 |
| 2005 | 15277 | 17 | 10 | 2 | 4 | 23594 | 81 | 62 | 13 | 4 | 14331 | 176 | 103 | 49 | 8 |
| 2006 | 15508 | 18 | 15 | 1 | 2 | 26863 | 93 | 74 | 10 | 4 | 14476 | 174 | 116 | 41 | 8 |
| 2007 | 11259 | 23 | 16 | 3 | 1 | 21198 | 57 | 42 | 7 | 4 | 15330 | 115 | 79 | 34 | 0 |
| 2008 | 11765 | 14 | 8 | 2 | 2 | 21619 | 60 | 44 | 6 | 9 | 16222 | 114 | 86 | 12 | 2 |
| 2009 | 43078 | 64 | 39 | 8 | 7 | 92084 | 193 | 128 | 30 | 14 | 85309 | 495 | 338 | 106 | 28 |
| 2010 | 45237 | 49 | 31 | 6 | 5 | 96840 | 200 | 124 | 18 | 32 | 89408 | 485 | 345 | 91 | 22 |
| 2011 | 45416 | 64 | 41 | 7 | 8 | 95815 | 215 | 138 | 16 | 42 | 89369 | 485 | 334 | 106 | 20 |
| 2012 | 49282 | 54 | 38 | 5 | 8 | 99025 | 157 | 94 | 20 | 27 | 92937 | 431 | 303 | 91 | 13 |
| 2013 | 47588 | 34 | 19 | 8 | 3 | 95578 | 97 | 68 | 5 | 14 | 93611 | 392 | 263 | 89 | 20 |
| 2014 | 46775 | 31 | 19 | 2 | 8 | 91551 | 140 | 95 | 9 | 21 | 93385 | 433 | 309 | 74 | 18 |
| 2015 | 50773 | 25 | 17 | 2 | 6 | 85268 | 79 | 49 | 10 | 16 | 91602 | 345 | 235 | 71 | 20 |
