# Supplementary material for: Communicable disease mortality trends and characteristics of infants in rural China, 1996–2015
Source: BMC Public Health. 2020 Apr 6;20:455. doi: 10.1186/s12889-020-08486-y (PMC7137429; doi:10.1186/s12889-020-08486-y)
Supplement: Supplementary file 3 — Additional file 3:Table S2. The comparison of communicable disease-specific IMR between 123 and 334 surveillance sites. [file 12889_2020_8486_MOESM3_ESM.docx]

Table S2. The comparison of communicable disease-specific IMR between 123 and 334 surveillance sites.

| Year | 123 surveillance sites | | |  | 334 surveillance sites | | |
| --- | --- | --- | --- | --- | --- | --- | --- |
|  | CD-IMR  (per 100000 live births) |  | 95%CI |  | CD-IMR  (per 100000 live births) |  | 95%CI |
| 2009 | 296.5 |  | (251.8, 301.6) |  | 298.2 |  | (275.9, 299.4) |
| 2010 | 268.0 |  | (225.8, 272.9) |  | 273.7 |  | (252.8, 274.8) |
| 2011 | 280.8 |  | (237.5, 285.8) |  | 290.8 |  | (269.2, 291.9) |
| 2012 | 207.8 |  | (171.4, 212.6) |  | 231.6 |  | (212.8, 232.6) |
| 2013 | 195.4 |  | (159.8, 200.3) |  | 183.8 |  | (167.0, 184.9) |
| 2014 | 226.1 |  | (187.8, 231.0) |  | 217.9 |  | (199.4, 219.1) |
| 2015 | 123.8 |  | (96.8, 128.4) |  | 162.8 |  | (146.7, 164.0) |
